# Legal Framework and Retirement Policies in Serbia from 1990 to 2016 – Gendered Perspective

**DOI:** 10.3389/fpubh.2016.00208

**Published:** 2016-09-27

**Authors:** Olivera Milovanovic, Svetlana Radevic, Mirjana Jovanovic

**Affiliations:** ^1^Department of Pharmacy, Faculty of Medical Sciences, University of Kragujevac, Kragujevac, Serbia; ^2^Department of Social Medicine, Faculty of Medical Sciences, University of Kragujevac, Kragujevac, Serbia; ^3^Department of Psychiatry, Faculty of Medical Sciences, University of Kragujevac, Kragujevac, Serbia; ^4^Psychiatry Clinic, University Clinical Center Kragujevac, Kragujevac, Serbia

**Keywords:** retirement policies, gender differences, legal framework, pension and disability insurance, education

## Abstract

Retirement policy is an unavoidable factor for the economic and social stability of the state. In our country, Serbia, the total number of pensioners in 2015 was almost 1.8 million, which is higher in comparison to the time period from 2002 to 2014. According to increased number of pensioners, pension reforms are a crucial step of economic stability for less developed country, such as Serbia. The first step in this question in Serbia was made in 2001, and this change was referred to the raise in the retirement age. Next step was made in 2003 and involved wider ranges of changes than the previous step. Another change in this field was followed by 2005, and it was related to age limit for retirement, which was supposed to increase for 6 months each year during the period from 2008 to 2011, while another change was a gradual pension adjustment. The last step in this road of pension reforms in Serbia has become with adopted Law on Pension and Disability Insurance that entered into force in January 2015, when age limit for retirement was extended for both genders, whereby it is clear that the aim of this measure is to equalize years of service for genders by 2023 and age limit by 2032 when 65 years will be the age limit.

## Population Aging and Retirement Processes in Serbia

Population aging causes imbalance in labor market, changes significantly the demand of social services, jeopardizes the functioning of a pension system, and disrupts the distribution of welfare between generations (1). The citizens of Serbia are among the oldest in the world (average age is almost 42 years) (2, 3). More than one fifth of the citizens are more than 65 years old, and there are almost as many people aged from 50 to 65. Loss of employment, rising unemployment, unregistered (illegal) work, more insecure working conditions, and the violations of the Labor Law and the Law on Safety and Health at Work in Serbia markedly affect older workers, aged from 50 to 65 who have lost their jobs or are in great risk of losing employment (4).

Pension policy is an unavoidable factor for the economic and social stability of the state, and its implications become especially prominent in countries in transition or less developed countries, such as Serbia (5). The decrease in the number of working age population and the increase in the number of retired people lead to the collapse of pension and disability system of a state and create the need for continuous efforts and reforms in this field in order to achieve a compromise solution both for individuals and a society and to accomplish long-term economic stability (6).

In most countries, pension system is organized as a form of obligatory insurance, and in this way, a country can support individuals when they are not capable of working, thus providing material security and sources for satisfying their basic needs. Another purpose of obligatory insurance is to disable the occurrence of poverty among older population (7). According to the Statistical report of Republic Fund for Pension and Disability Insurance, in Serbia, the total number of pensioners in 2015 was almost 1.8 million, which is higher in comparison to the time period from 2002 to 2014 (8).

The question of pension reforms has been the priority of the Government working within the different political settings in Serbia over the last two decades. Population aging phenomenon, insufficient birth rate, and a consequent decrease in number of working age population, as well as longer life expectancy create additional pressure on the social system (9).

The first step in Serbia in this regard was made in 2001 when Law on Pension and Disability Insurance was changed and came into force at the beginning of the next year. The adopted changes were ambiguous as they referred to the raise in the retirement age: for men, it was raised from initial 60 to 63 life years and for women from 55 to 58 life years. Another aspect of these changes is the method of pension adjustments through the so-called “Swiss-formula.” Phenomenon of “Swiss-formula” according to the Law from 2003 implied pension adjustment in payments at the quarterly level according to the values of living costs and average earnings of employed population during the previous 3 months (10, 11).

The initiated changes were continued with the new Law on Pension and Disability Insurance, which became effective in April 2003 by involving clauses about disability definition, cancelation of rights on the basis of remaining working ability, and revision of disability pension. In compliance with the mentioned law changes, certain modifications were made concerning the years of service. Instead of calculating pensions based on the best 10 years of service, now they were calculated based on all years of service (10).

The next step in the process of reform occurred at the end of 2005 and came into force in January 2006. One of the changes in this step referred to the age limit for retirement, which was supposed to increase for 6 months each year during the period from 2008 to 2011 and which consequently changed the age limits for family pensions. After these changes, age limit for retirement was 60 years of life for females and 65 years for males. Another change was a gradual pension adjustment (from 2006 to 2009) when a 4-year pension adjustment with “Swiss-formula” was replaced with 2-year pension adjustment based on the life costs. According to this law, an extramural adjustment was planned for January of the following year if the average pension in the past year had been lower than 60% of Serbian average salary without taxes and contributions (11). Under this law clausal, a pension adjustment of 11% was performed in January 2008, and in October, the additional adjustment of 10% in addition to a regular one was also made. After this, pension values were frozen. This political measure had been applied until the end of 2008. During the same year, the minimal value of pension was determined at 25% of an average salary of an employed person during 2005. This event was followed by a pension adjustment according to the principle of harmonization with other pensions with recommendations that an extramural adjustment should be performed on January 1 of the following year if pension value had been lower than 20% of average salary.

Funds consolidation was also included in this reforming step, and its enforcement and further reformation of pension process were made possible only after Serbia got $25 million of loan from the World Bank (12). Concurrently, the Law on Public Debt was defined and adopted which established a regular rhythm of pension payments to insured persons, i.e., they were paid this month for the previous one (10–13).

## Pension Structure in Serbia and Gender Differences

The data about pension structure in Serbia from 2008 reveal that the highest percentage went to age pensions, almost a half, while the rest went to disability and family pensions. In the mentioned pension structure, family pensions make one fifth of the total number, which can be explained by possibility of transition from age pension to family pension when the insured person is woman. Age limit for retirement in 2008 was more favorable for woman than for man: it was 58.5 years for women versus 63.5 years for men.

Based on the data from January 2016, an interesting observation can be made that pension structure has identical distribution, as 8 years ago, the highest percentage had gone to the age pension, even 61.38%, while family pension still held a fifth of the total number that is 20.68% of the pensioned people (14).

In accordance with the current Government policy in the Republic of Serbia and adopted Law on Pension and Disability Insurance that entered into force in January 2015, age limit for retirement was regulated and it will be successively extended for both women and men, whereby it is clear that the aim of this measure is to equalize years of service for genders by 2023 and age limit for genders by 2032 when both men and women could retire at the age of 65. Article number 19 of the mentioned Law indicates that a person has the right to age pension when he/she reaches 65 years of life and minimum 15 years of working service or only 45 years of working service (10). From the abovementioned, it can be concluded that besides age limit which is one of the conditions for age retirement, years of service can be a crucial factor for pension achievement. Although the requirements in terms of years of service are now the same, according to the previous laws they differed – for women, the limit had been 35 years of service at first and was then increased to 38, while for men, it was 40 years.

Under the present legislation, in 2016, for regular pension, women need 61 years of life and men 65 years, whereby the conditions are getting unfavorable for women since their age limit is supposed to increase annually until age limit for both genders become equal by 2032. Successive changes of age limit in period from 2014 to 2020 are carried out by adding 6 months to the previous value, while in the period from 2021 to 2023, 2 months will be added (10). This law also plans and changes the criteria for going to earlier retirement for both men and women in period from 2015 to 2023 in order to achieve a partial equalization of conditions for both genders (Table 1).

**Table 1:** **Requirements for going to early retirement Article 19v from Law on Pension and Disability Insurance (9)**.

Calendar year	Men	Women
2015	40 years of service and minimum 55 years of age	36 years and 4 months of work experience and a minimum of 54 years and 4 months of age
2016	40 years of service and minimum 55 years and 8 months of age	37 years of service and a minimum age of 55 years
2017	40 years of service and minimum 56 years and 4 months of age	37 years and 6 months of service and a minimum age of 55 years and 8 months of age
2018	40 years of service and minimum 57 years of age	38 years of service and a minimum age of 56 years and 4 months
2019	40 years of service and minimum 57 years and 8 months of age	38 years and 6 months of service and a minimum age of 57 years
2020	40 years of service and minimum 58 years and 4 months of age	39 years of service and a minimum age of 57 years and 8 months
2021	40 years of service and minimum 59 years of age	39 years and 4 months of service and minimum 58 years and 4 months of age
2022	40 years of service and minimum 59 years and 6 months of age	39 years and 4 months of service and minimum 59 years of age
2023	40 years of service and minimum 60 years of age	40 years of service and minimum 59 years and 6 months of age

The integration of issues related to gender specificity into a legal framework, national strategies, and plans have been current in the last few years based on the political aspirations toward European Union. Gender differences are observed in the areas of education, health, employment, and pension system (15). Based on Labour Force Questionnaire conducted by Republic Institute for Statistics (2008–2014), it has been revealed that there were no significant differences in participation of women and men in educational processes in Serbia (16):

- Both genders, on average, have the same achieved level of education.
- There is a higher level of participation of woman in the low levels of education (primary school or less).
- The characteristic of the older age group (from 55 to 64 years) in rural areas is that most women work in agriculture or are economically inactive (trends suggest that this gap will be reduced in future).

It was also noted that there is greater participation of women with higher or university education, primarily in the middle age group (from 25 to 54 years old), both in employed and unemployed group. The gap in education is also increasing in this age group in respect to gender in favor of women. Besides, the data have shown that women are less likely than men to leave education earlier.

Men and women job framework has been changed during the past few decades, and it is clear that there is an increasing trend in woman employment. Despite this, women are still unequal users of pension insurance, generally speaking from the point of lower education level and consequently lower income during the working experiences.

### Employment and Gender Differences

When they are on the labor market, women with the same educational level as men have equal chances of being employed. Higher female unemployment can be explained by differences in the characteristics of the labor market (greater work experience of men). Women are facing the barriers when they enter labor market, which results in their lower employment. The differences are most visible in older age groups (from 55 to 64 years) as well as in the group of those with low education and in rural settlements. Lower activity of women is not only associated with childcare and gender roles but also with stronger effects of receiving social transfers and pensions at household activity. The concept of work has gone through many transformations; contracts are undefined or unfavorable for workers; there are atypical forms of employment, and all this just affects women and brings in the question of equality at the labor market (17).

Danish sociologist Goste Esping-Andersen, in his research from 2002, has noted that there was an increasing polarization of the unemployed people based on gender, age, and qualifications. Assuredly, most vulnerable families were those whose members had insecure or temporary employment, single parents, and marginal groups, which are all mostly female (18). In addition, Serbia has one of the highest unemployment rates in Europe. Women’s position in the labor market in Serbia shows that typically female jobs are losing the race in the market game where there are still gender-specific jobs. Sociological studies in this area suggest that old misconceptions and ineffective model of emancipation of women in the former socialist society are transferred to the modern stage of transitional movement toward capitalism (19–21).

Age discrimination (ageism) is a phenomenon that is increasingly evident in modern times (22), when the age of workers appears to be a reason for release or for not hiring. Usually, we talk about discrimination in the four areas of the labor market: (1) loss of employment (or premature loss of employment) – when a company goes through restructuration of production processes and has to reduce the number of employees before the release of older workers; (2) difficulty in employment – when younger candidates are employed rather than older ones, i.e., those being 40, 50, or even older; (3) exclusion of vocational training – older workers, both employed and unemployed, have more difficulties in passing the selection for training programs; vocational training programs targeting at older workers are very rare; and (4) retirement – older workers are required to retire once they meet the legal requirements, and if they meet certain conditions, they get certain kinds of “support.”

Women have most often been employed in poorly paid jobs, working roles that leave plenty of time for family and raising children. Although the legal framework defines the amount of earnings for men and women to be equal as one of the most important aspects of female equality at the labor market, women are still paid less than men. This can be explained with the fact that women are employed at job positions, which are less prestigious and provide less financial means. There are not any direct statistical data on the amount of income earned by women and men in Serbia, but it can be indirectly concluded that the activities in which women are employed, such as jobs in the textile industry, public administration, education, or social protection, are on the list of less-paid jobs. With low earnings that are made by woman in Serbia, they are at similar position like women from other post-socialist countries, as well as from neighboring countries. Women earn less than men even in the most developed countries of the European Union. Earnings of women were 17.4% lower than men’s earnings when considered the average of the European Union (EU-27) for 2007 (23).

Up to now, age limit for women according to the Law was more convenient for women than for men, but with the beginning of the reform process, this advantage began to decline slowly. Women had an advantage in terms of calculating years of service when years of service were increased by 15% until one reached 40 years of service. In other words, if a female had had 35 years of service, she would have got additional 15%, but if she had had from 35 to 40 years of service, it would have been counted as if she had had 40 years of service. It is evident that in the second case, the increase was lower than with the first option. On the other hand, if women had had more than 40 years of service, no additional time would have been added and the advantage women had in previous cases over men would vanish.

The aspect of family pension is legally regulated in Serbia so that a person who succeeds the pension is entitled to 70% of the pension value of the deceased insured person, and there was a slight bias toward female gender so that after the reform, the retirement age limit in 2011 was 50 for women and 55 for men. Under the new Law, age limit as condition for family pension is increased for 3 years, so for woman, it was raised from 50 to 53 years, while for men, it went from 55 to 58 years. Predicted period for execution of those reforms is from 2014 to 2017 (10, 24).

In addition to the aforementioned factors that may affect status of women in the pension system, it should be noted that there is a framework for maternity absence or childcare absence, which is very important element of gender equality, otherwise it would be very difficult to meet the conditions for pension in comparison to man. During the period of pregnancy maintenance, our country paid 65% of previous earnings of person in period from 2005 to 2013, while from January 2014, this value was raised to 100% which reflects on pensions. Important note is that women who have given birth to three or more children get 2 years of working experiences, but these years are not included in the number of years needed for age retirement but are included in the calculations of personal points which reflects on the amount of pensions (10).

## Future Directions of Movement

It is necessary to make a broader insight and systematic detection of the position of women in socioeconomic context and improve knowledge of sociodemographic status, especially for vulnerable groups, such as unemployed women, new mothers, people with severe and/or chronic diseases, single mothers, people in older age, etc. More detailed insight into the health-care system is necessary, i.e., better understanding of adjustment mechanisms of social and health responses to the specific needs of women (25). In accordance with the abovementioned, one of the priorities is to develop tools that would help social services and health-care professionals to efficiently act in specific situations (26).

### Political Level

Influence of the EU accession process to the creation of a regulatory framework in the field of gender equality is present in Table 2.

**Table 2:** **The impact of the EU accession process to the creation of a regulatory framework in the field of gender equality**.

Transposition of EU legalization in the field of gender equality in the national legal framework	Law on Gender Equality (2009) Law on Prohibition of Discrimination (2009) Family Law (2011) Labour Act (2014)
The establishment of national institutions for the promotion of gender equality	Coordinating body for gender equality Gender Equality Council Parliamentary Committee for Human and Minority Rights and Gender Equality Equality Commissioner Deputy Ombudsman for Gender Equality
Positive impact on the mobilization of the women’s movement	EU encourages the creation and development of governmental and non-governmental organizations dealing with gender issues

## Redistribution from Men to Women

Redistribution from men to women is a normal advent in the most systems of pension, and their implementation is justified by the appearance of longer lifetime of women in comparison to men and thus longer time for retirement using (27). This phenomenon is not justified to some extent in our country since the demographic data are not entirely consistent with this statement (7, 11). Demographic analysis has shown that women in Serbia live shorter than women in other European countries, and this fact could slow down recommendations related to pension reforms adoption from other countries (11).

Conclusion from the current data is that we need full implementation of the existing regulatory framework which will ensure gender equality, but it is also necessary to adopt measure and policy recommendations that will encourage activity of women who have low earning capacity (low education and little or no work experience). Women’s leadership in politics and the economy should be promoted in order to encourage changes in attitudes in terms of greater participation of women in all spheres of life.

## Author Contributions

Exploration of review of literature and manuscript write up. All the above listed authors have made substantial, direct, and intellectual contribution to the work and approved it for publication.

## Conflict of Interest Statement

The authors declare that the research was conducted in the absence of any commercial or financial relationships that could be construed as a potential conflict of interest. The reviewer AK and handling editor declared their shared affiliation, and the handling editor states that the process nevertheless met the standards of a fair and objective review.
